# PLAC8 Expression Regulates Trophoblast Invasion and Conversion into an Endothelial Phenotype (eEVT)

**DOI:** 10.3390/ijms26115371

**Published:** 2025-06-04

**Authors:** Laura J. Barragán-Zúñiga, Rodrigo Escalona-Rivano, Catalina Cordero-Tirado, Martha Sosa-Macías, Ivo Carrasco-Wong, Jaime Gutiérrez, Carlos Galaviz-Hernandez

**Affiliations:** 1 Instituto Politécnico Nacional, CIIDIR-Durango, Durango 34220, Mexico; ljbarraganz@gmail.com (L.J.B.-Z.); msosam@ipn.mx (M.S.-M.); 2 Centro Estatal de Cancerología, Secretaria de Salud de Durango, Durango 34000, Mexico; 3 Faculty of Medicine and Science, Universidad San Sebastián, Santiago 7510602, Chile; rs.escalona.rivano@gmail.com (R.E.-R.); ccorderot@correo.uss.cl (C.C.-T.); ivo.carrasco@uss.cl (I.C.-W.)

**Keywords:** PLAC8, trophoblast invasion, endothelial-like differentiation, preeclampsia

## Abstract

PLAC8, expressed by interstitial extravillous trophoblasts (iEVTs), plays a crucial role in trophoblast invasion, differentiation, and immunotolerance. Its dysregulation may contribute to pregnancy complications, such as preeclampsia. This study investigates the role of PLAC8 in trophoblast invasiveness and endothelial-like differentiation under different oxygen tensions. Swan-71 cells were transiently transfected with *PLAC8* overexpression or knockdown plasmids. Invasion was assessed using Matrigel-coated transwells, endothelial-like differentiation through tube formation assays, and vasculogenic marker expression (VEGF, PGF, ANGPT2) by RT-PCR. Hypoxia experiments were performed at different oxygen conditions. *PLAC8* overexpression enhanced trophoblast invasion but reduced endothelial-like differentiation, downregulating VEGF and PGF while upregulating ANGPT2. Hypoxia increased PLAC8 expression, indicating oxygen tension as a regulatory factor. PLAC8 manipulation did not affect cell viability. PLAC8 modulates trophoblast behavior by promoting invasion while inhibiting endothelial-like differentiation. Its regulation of vasculogenic and angiogenic factors suggests a critical role in placental homeostasis and potential relevance to pregnancy disorders, such as preeclampsia.

## 1. Introduction

The placenta is a temporal and dynamic organ that plays a critical role during pregnancy, as it is vital for the survival and health of the embryo and the mother [1]. Different diseases or syndromes in pregnancy have their origin in the placenta [2], with the most severe being preeclampsia (PE) [3], which is associated with major maternal and fetal perinatal complications. The pathophysiological mechanism of PE is unclear, but the evidence indicates its origin in the inefficient remodeling of the uterine spiral arteries (SAs) during placentation [2]. This remodeling process requires a group of fetal cells derived from the blastocyst, known as trophoblast cells. A subgroup of them, known as extravillous trophoblast (EVT) interstitially invade (iEVT) the maternal decidua towards the SAs [4]. Once there, iEVTs replace the SA endothelial cells via the acquisition of an endothelial-like phenotype (eEVT), remodeling the SAs to increase the maternal blood flow to the placenta, thus providing the blood flow required by the fetus [5,6].

In PE, both the invasion of iEVT and their differentiation to eEVTs are affected, emerging as causes of the pathology [7]. Transcriptomic and proteomic analyses allowed the identification of different genes and proteins which have been associated with such processes [8]. Among them is the placenta-specific protein 8 (PLAC8), which is specifically expressed by iEVTs but downregulated in endovascular extravillous trophoblasts (enEVTs) associated with uterine vessels, as demonstrated by Chang et al. [9], in human placenta as well as in primary cultures of human trophoblast. Moreover, they discovered that PLAC8 promotes the invasion and migration of iEVT and that its expression is induced by culturing them under low oxygen conditions, which is consistent with the evidence that low oxygen tension, a common condition during early placenta development [9], promotes the differentiation of CTB to iEVT and the induction of their invasiveness [10]. However, the participation of PLAC8 in the control of iEVT differentiation toward eEVTs has not been addressed. The same authors found that PLAC8 expression is higher in iEVTs from preeclamptic placentas compared to those from normal pregnancies [9], which was explained by the chronic hypoxia commonly observed in preeclamptic placentas [11,12]. However, this is inconsistent with the widely accepted observation that, in preeclamptic placentas, the differentiation and subsequent invasion of iEVTs are reduced [13], which would suggest a decreased expression of PLAC8.

Transforming growth factor-β1 (TGF-β1) is a factor that participates in the regulation of different cellular processes, including cell proliferation, apoptosis, and migration in different cells [14,15,16] as well as in the regulation of angiogenesis and vascular development [8,17,18,19]. In trophoblast cells, TGF-β1 acts as an invasion inhibitor [20,21]. Moreover, TGF-β1 signaling is augmented in PE patients compared to normal pregnancies [22,23]. Thus, TGF-β1 appears to be a potential positive regulator of the expression of PLAC8 in EVT.

The aim of this study is to evaluate the role of PLAC8 and its regulation by oxygen tension in first-trimester human trophoblast cell lines as a crucial factor affecting EVT invasiveness and differentiation into an endothelial-like phenotype. Furthermore, it seeks to examine TGF-β1 signaling as a potential regulator of PLAC8 expression.

## 2. Results

### 2.1. The Knockdown and Overexpression of PLAC8 Have No Effect on Cell Viability

**Figure 1:**
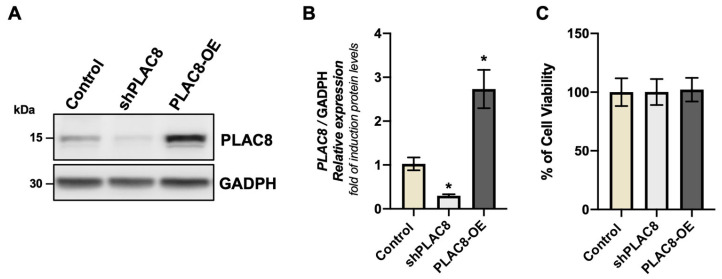
PLAC8 expression and viability in Swan-71 cells. (**A**) Representative Western blot for PLAC8 silencing and overexpression; GADPH was used as a loading control. (**B**) PLAC8/GADPH protein densitometry. (**C**) Cell viability performed with MTT assay. Swan-71 cells transfected with an empty pRP vector (Control), shRNA against human PLAC8 (sh*PLAC8*), and pRP vector containing the *PLAC8* sequence (PLAC8-OE) (see Section 4). Data are mean ± SD *p* < 0.05 (*).

In order to evaluate the effect of PLAC8 expression on cell viability of extravillous trophoblast cells, we took advantage of the Swan-71 trophoblast, a cell line derived from a human first-trimester placentas. The expression of the PLAC8 protein was knocked down by the expression of a shRNA against the mRNA of *PLAC8* (sh*PLAC8*) and overexpressed by the transfection of an expression vector which drives the expression of *PLAC8* under the control of a constitutive promoter (*PLAC8*-OE). Western blotting, along with its corresponding densitometric analysis (Figure 1A,B), illustrates the levels of the PLAC8 protein abundance in cell extracts of the transfected with the indicated constructs. The induced expression or knockdown of PLAC8 protein did not impact the viability of Swan-71 cells, as demonstrated in Figure 1C.

### 2.2. PLAC8 Promotes Invasion and Responds to Oxygen and TGF-β1 Signals in EVT Cells

**Figure 2:**
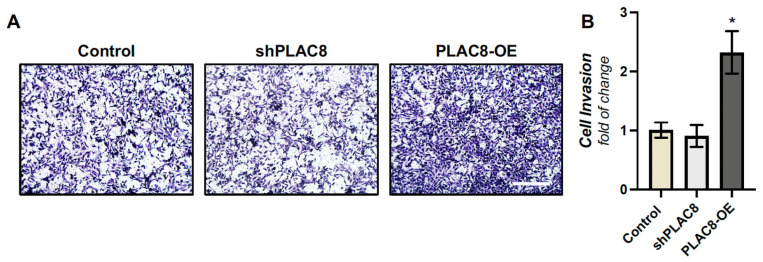
Participation of PLAC8 in the invasion of Swan-71 cells. (**A**) Representative photographs of Swan-71 cells transfected with an empty pRP vector (Control), shRNA against *PLAC8* (sh*PLAC8*), or pRP vector containing the *PLAC8* sequence (*PLAC8*-OE) that migrated in transwells with polycarbonate filters (8 mm pore) (see the Section 4). (**B**) Absorbance (595 nm) for crystal violet staining present in cell lysates. 10× magnification. Data are mean ± SD *p* < 0.05 (*).

**Figure 3:**
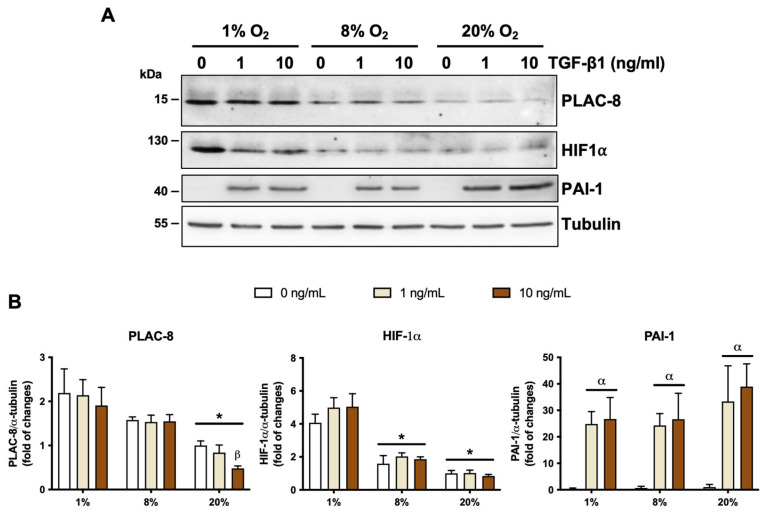
Effect of oxygen and TGF-β1 on PLAC8 protein expression. (**A**) Representative Western blot for PLAC8, HIF-1α and PAI-1 protein expression. The Western blot shows the expression of PLAC8, HIF-1a and PAI-1 proteins under TGF-β1 treatment and various O_2_ conditions. α-tubulin was used as a loading control. Asterisks (*) indicate significant differences between study groups (*p* < 0.05) according to Tukey’s test. (**B**) Densitometry of PLAC8 and HIF Proteins. White bars represent vehicle (control), yellow bars represent TGF-β1 1 nM, and brown bars represent TGF-β1 10 nM. Oxygen conditions are set at 1%, 8%, or 20% (see the Section 4 for details).

To validate our EVT cell line as a model in this study, we aimed to evaluate two effects related to PLAC8 in our system which were previously described in primary cultures of human trophoblasts [9], namely (i) the enhanced invasion observed with *PLAC8* overexpression and (ii) the upregulation of *PLAC8* expression under low oxygen culture conditions. We evidenced that the transient overexpression of *PLAC8* in Swan-71 trophoblasts resulted in more than twofold increase in invasiveness compared to control trophoblast cells in a transwell invasion assay, while its knockdown had no effect (Figure 2A,B). We also observed that PLAC8 expression progressively increased when the cells were cultured at 8% and 1% O_2_ compared to 20% O_2_ (Figure 3A,B). HIF1-α protein abundancy was determined as a control of hypoxia, which was induced at 1% O_2_ compared to 8% and 20% O_2_, and at 8% compared to 20% O_2_ (Figure 2A,B). These results indicate that Swan-71 trophoblasts are a good model for the study.

We also evaluated the effect of TGF-β1 on PLAC8 expression (Figure 3) under three oxygen conditions (1%, 8%, and 20% O_2_). We observed that TGF-β1 has no effect on PLAC8 abundance at 1% and 8% O_2_, despite the fact that the cells effectively responded to the ligand, as evidenced by the expression of PAI-1, a classic TGF-β1 response gene. However, in cells cultured at 20% O_2_, TGF-β1 at 10 ng/mL reduces PLAC8 protein abundance by approximately 50%, which was also associated with PAI-1 expression. No difference in PAI-1 expression in response to TGF-β1 was observed among the three different oxygen conditions.

### 2.3. PLAC8 Expression Is Induced in Trophoblast Cells Stimulated to Acquire an Endothelial-like Phenotype, Acting as a Negative Regulator of This Process

**Figure 4:**
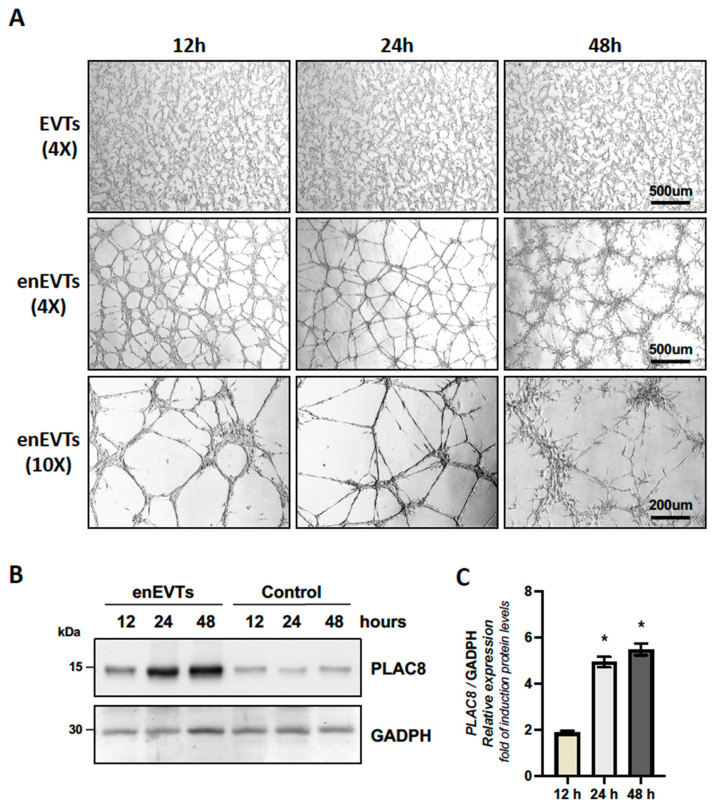
Swan-71 trophoblast cells form tube-like structures indicative of endothelial-like differentiation (eEVTs) at 12, 24, and 48 h post-seeding on Matrigel (**A**). Western blot analysis shows increased PLAC8 expression at 12 h, with levels stabilizing at 24 and 48 h in enEVTs (**B**,**C**). * significant differences of 24 h and 48 h regarding 12 h.

To evaluate the role of PLAC8 in the organization of trophoblast cells stimulated to acquire an endothelial-like phenotype, we performed a classical angiogenesis assay on Matrigel. As shown in Figure 4, Swan-71 trophoblast cells progressively formed tube-like structures, acquiring an endothelial-like phenotype (eEVTs) compared to trophoblast cells cultured without Matrigel (EVT) at the same the western blot analysis presented in the Figure 4B and its quantification in the Figure 4C, demonstrates that PLAC8 expression increases significantly at 12 h, reaching plateau at 24 and 48 h after the induction of the endothelial-like phenotype. This result suggest that PLAC8 may play a regulatory role in this process.

**Figure 5:**
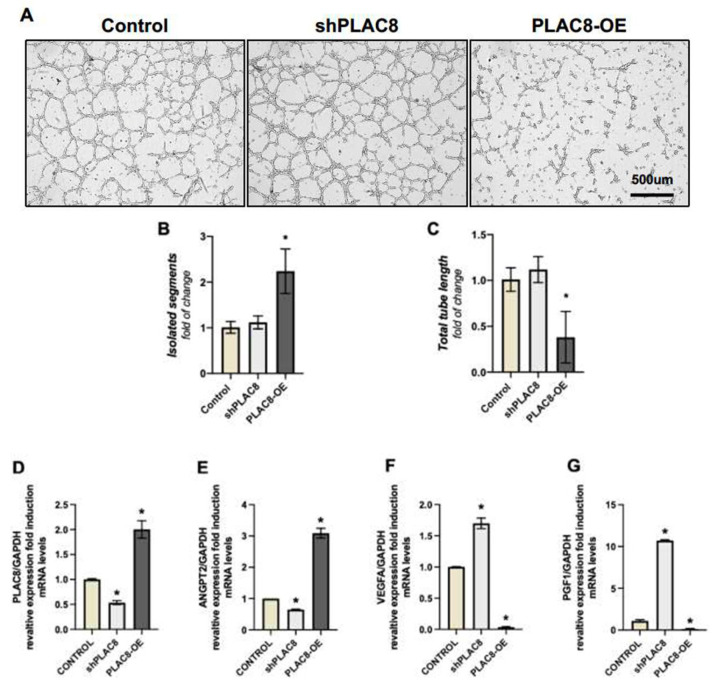
PLAC8 expression and its effect on endothelial-like trophoblast organization and angiogenesis-related gene expression. (**A**) Representative images of endothelial-like trophoblasts (eEVTs) under the following three conditions: control cells transfected with an empty pRP vector, cells with downregulated *PLAC8* (sh*PLAC8*), and cells with upregulated *PLAC8* (*PLAC8*-OE). (**B**) Analysis of unbranched segments of eEVTs in each *PLAC8* expression condition. (**C**) Total tube length of eEVTs under each condition. (**D**–**G**) qPCR analysis of angiogenesis-related gene expression in response to *PLAC8* knockdown and overexpression. (**E**) *ANGPT2* mRNA levels, (**F**) mRNA levels, *VEGFA* mRNA levels, abnd (**G**) *PGF1* mRNA levels. Data are presented as mean ± SD, with significance set at *p* < 0.05 (*).

To evaluate this hypothesis, we examined the effects of the knockdown and overexpression of *PLAC8* (Figure 5A). The knockdown of *PLAC8* had no effect on the formation of tubular structures compared to control cells. However, *PLAC8* overexpression resulted in shorter, unconnected segments with no branching. This led to an increase in unbranched segments that were unable to form proper tubular structures (Figure 5B). Furthermore, the few tubes that did form were shorter in length (Figure 5C). We also assessed the expression of genes traditionally associated with angiogenesis control in response to the knockdown and overexpression of *PLAC8* using qPCR. Figure 5D illustrates the effects of the silencing and overexpression vectors at the mRNA level. *PLAC8* overexpression induces an increase in *ANGPT2* mRNA levels (Figure 5E), which is associated with the suppression of new vessel formation, while simultaneously reducing the expression of *PGF1* and *VEGFA*, two genes that promote angiogenesis (24) (Figure 5F,G). Conversely, silencing of *PLAC8* decreases *ANGPT2* expression (Figure 5E) and induces *PGF1* and *VEGFA* (Figure 5F,G).

### 2.4. The Invasion and Differentiation of Trophoblasts Under Varying Oxygen Conditions Are Partially Regulated by the Differential Expression of PLAC8

**Figure 6:**
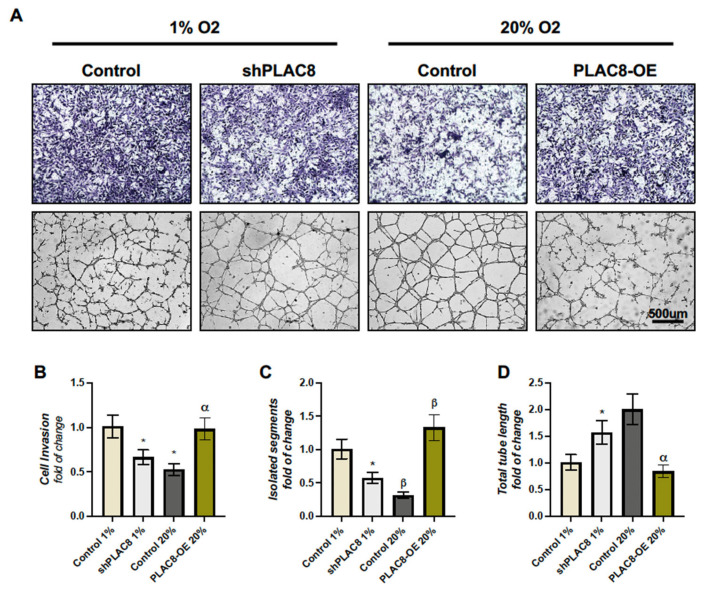
The role of PLAC8 in regulating trophoblast invasion and differentiation under varying oxygen conditions. Swan-71 trophoblasts were cultured under low (1% O_2_) and high (20% O_2_) oxygen levels to assess their invasion capacity and the ability to form tube-like structures. Low oxygen (1% O_2_) increased invasion at the expense of tube formation, while high oxygen (20% O_2_) enhanced differentiation but reduced invasion (**A**). (**B**) Analysis of cell invasion; (**C**) unbranched segments of enEVTs in each condition. (**D**) Total tube length of enEVTs under each condition. Data are presented as mean ± SD, with significance set at *p* < 0.05 (*). * means significantly different, while β indicates significant difference from all other bars. α indicates significant difference from shRNA at 1% O_2_ and control at 20% O_2_.

The previous results suggest that PLAC8 functions as an inducer of invasion and a repressor of the differentiation of extravillous trophoblasts towards an endothelial-like phenotype. Additionally, considering that PLAC8 expression is induced by low oxygen concentrations, we assessed whether low and high oxygen levels influence the processes of invasion and the ability to form tube-like structures in a manner dependent on PLAC8 expression. Figure 6 demonstrates that Swan-71 trophoblasts cultured under low oxygen tensions (1% O_2_) enhance invasion at the expense of their ability to form tube-like structures compared to those cultured under high oxygen concentrations (20% O_2_), an effect that was diminished by the knockdown of *PLAC8* under low oxygen conditions. Conversely, the decreased invasion and enhanced differentiation observed at 20% O_2_ compared to 1% O_2_ were reversed by the overexpression of *PLAC8*. These results indicate that the behavior of trophoblasts under varying oxygen conditions is, in part, regulated by the differential expression of PLAC8.

## 3. Discussion

Adequate placental perfusion depends on trophoblast invasion and remodeling of the uteroplacental arteries, which require an exquisite regulation of the invasion process itself, the immune tolerance between the fetal trophoblast invading the maternal tissue, and the commitment and differentiation of iEVTs upon their arrival at the spiral arteries, where they replace the maternal smooth muscle and endothelial cells [24]. Reduced placental perfusion is the hallmark of severe pregnancy-specific syndromes, such as preeclampsia and intrauterine growth restriction [25]. Thus, great efforts have been made to define the regulatory mechanisms governing this intricate and vital process. PLAC8 is a specific marker of iEVTs in the human placenta [9,26] and has been studied and characterized in the process of trophoblastic invasion and differentiation [9] and as a regulator of the immunotolerance activity in placental development and cancer [27,28].

In brief, our findings show that *PLAC8* overexpression in trophoblast cells (Swan-71 cells) stimulates their invasiveness and inhibits the expression of pro-angiogenic mediators as well the formation of tubular structures by EVT cells, which is reminiscent of its role in endothelial-like differentiation, which is essential for the proper remodeling of spiral arteries (SAs).

We demonstrated, in Swan71 cells (a human first-trimester extravillous trophoblast cell line), that *PLAC8* knockdown or overexpression has no effect on cell viability. It was previously identified that PLAC8 as an exclusive marker for interstitial-type extravillous trophoblasts (iEVTs), suggesting its potential involvement in cell migration and differentiation [9]. Furthermore, the expression of PLAC8 is induced under low O_2_ culture conditions, a factor recognized for its significant role in influencing trophoblast invasion and differentiation [29,30]. Although these results provide valuable insights, it is important to note that they were obtained using the Swan-71 cell line, which, while validated and widely used, may not fully recapitulate the cellular diversity and dynamic microenvironment of the placenta in vivo.

Since O_2_ concentrations negatively impact the invasiveness of trophoblast, i.e., under low O_2_ concentrations the invasion is favored [31], we evaluated the effect of gain- and loss of function of *PLAC8* under normal (20%) and low O_2_ (1%) culture conditions. We validated previous studies showing that *PLAC8* overexpression promotes invasiveness [9,32]; however, in our study, *PLAC8* knockdown had no effect on EVT invasiveness at ambient O_2_ as previously described [9]. Interestingly, *PLAC8* knockdown effectively reduced trophoblast invasiveness only under low oxygen/pro-invasive culture conditions. We also confirmed previous results showing that low O_2_ culture conditions induce PLAC8 expression in EVT [9]. Thus, our results support the hypothesis that the increased invasion observed under low O_2_ is at least partly PLAC8-dependent. This hypothesis is also in agreement with the positive O_2_ gradient that EVTs encounter during the invasion from the implantation site to the spiral arteries [33]. Thus, it makes sense that when EVTs arrive at the spiral arteries, *PLAC8* expression is reduced, allowing the EVTs to reduce their invasiveness and initiate the spiral artery remodeling process. It would be interesting to evaluate whether, in conditions of hyperinvasive trophoblasts, as seen in placenta accreta, increta, and percreta [34,35], PLAC8 expression levels in the trophoblast fail to decrease, resulting in persistent, uncontrolled invasion. This idea requires further research.

In a previous study published by our working group [36], as well as by other groups [9], it was observed that *PLAC8* expression is increased in EVTs from preeclamptic placentas compared to those in placentas without preeclampsia. It has been proposed that chronic hypoxia, characteristic of preeclamptic placentas [37], explain the increased *PLAC8* in EVTs from preeclamptic placentas. This idea is somewhat controversial, given that shallow invasion of extravillous trophoblasts (EVTs) and the subsequent inadequate remodeling of spiral arteries are proposed as the origin of this syndrome [38]. However, it should be noted that this observation was made in placentas obtained at term [9], which are significantly affected by the syndrome. Thus, the increased expression of PLAC8 in extravillous trophoblasts (EVTs) from preeclamptic placentas could be attributed to various other factors or stimuli present in term placentas [37], potentially leading to heightened PLAC8 expression while simultaneously impacting their invasive capacity. Additionally, it is possible that EVTs upregulate *PLAC8* expression as an ‘emergency’ response to enhance invasiveness in an anti-invasive environment [39]. Therefore, further investigation into the expression and role of *PLAC8* in EVT invasion during the early stages of placental development is warranted. Although a direct experimental link to preeclampsia was not established in this study, previous reports have shown increased PLAC8 expression in placental tissue from preeclamptic pregnancies. It has been reported that PLAC8 expression is barely detectable in extravillous trophoblasts (EVTs) associated with remodeled uterine arteries [9], a finding consistent with the hypothesis that reduced PLAC8 expression is necessary to halt EVT invasion. In our study, we examined PLAC8 expression during EVT differentiation into an endothelial-like phenotype using a classical Matrigel angiogenesis assay at ambient O_2_ concentrations. In this assay, we observed that PLAC8 expression increased twofold after 12 h, reaching a plateau of nearly fivefold between 24 and 48 h after the initiation of the differentiation process, compared to non-differentiating EVTs at the same time points. This suggests that *PLAC8* plays a role in this process. Contrary to our expectations that PLAC8 would promote the acquisition of the endothelial phenotype by EVTs, overexpression of *PLAC8* was associated with a reduction in the expression of classical pro-angiogenic factors, such as *VEGF* and *PGF1*, and with the induction of the anti-angiogenic factor *ANGPT2*. Moreover, *PLAC8* overexpression notably reduced the formation of tubular structures, as evidenced by an increase in unbranched sections that lacked the capability to generate vascular-like structures. This suggests that *PLAC8* acts as a negative regulator of the endothelial-like differentiation process. The observed dual effect of PLAC8—enhancing invasion while reducing endothelial differentiation—may reflect an imbalance in trophoblast plasticity, potentially compromising proper spiral artery remodeling. Such a disruption has been implicated in the pathogenesis of placental disorders, including preeclampsia, where inadequate endothelial transformation and persistent pro-invasive signals coexist. In this context, it would be of interest, in future, to evaluate the expression of PLAC8 in trophoblasts under other conditions of abnormally invasive placenta, such as placenta percreta, accreta, and increta, where the placenta grows excessively deep into the uterine wall. Despite these findings, the knockdown of *PLAC8* did not exhibit the potentiating effect that one might expect in this assay. This finding underscores the existence of a narrow temporal window during which *PLAC8* expression is crucial for normal placental development. This proposal is supported by our results, which demonstrate that the formation of tubular structures by EVTs in the angiogenesis assay is significantly reduced under low O_2_ conditions, associated with increased *PLAC8* expression. Interestingly, under these low O_2_ conditions, the knockdown of *PLAC8* partially reversed the reduction in the formation of tubular-like structures.

This suggest that *PLAC8* plays a pivotal role in the initial stages of differentiation process which is also dependent on the stimuli provided O_2_ concentration.

Moreover, the seemingly contradictory result that PLAC8, an apparent inhibitor of endothelial-like differentiation, is upregulated in EVTs during their differentiation, might indicate a regulatory effect from maternal smooth muscle cells, endothelial cells, and/or immune cells on differentiating EVTs. In our assay, the EVTs organized themselves into tubular-like structures in the absence of preexisting vessels, which serves as a natural stimulus for EVT differentiation into the endothelial-like phenotype [40]. It is possible that smooth muscle or endothelial cells release soluble signals that reduce *PLAC8* expression in EVTs as they begin to incorporate into the spiral arteries. In this context, we found that TGF-β1, a recognized pro-angiogenic factor [17] produced by endothelial cells [41], reduces PLAC8 protein abundance in trophoblasts only when they are cultured under high O_2_ conditions.

In this study, Swan-71 cells were subjected to TGF-β1 treatment (as displayed in Figure 5), reducing the PLAC8 protein expression only under high O_2_ conditions. The alignment of these findings with the observed overexpression of PLAC8 under conditions of low oxygen tension (1%) suggests a direct response to hypoxia within first-trimester human trophoblast cells. Of note, when considering the diverse concentrations explored for TGF-β1, the highest concentration (10 nM) emerged as the most potent stimulator of PLAC8 expression. These findings establish an intriguing link to the previously noted escalation in inflammatory mediators, like TGF-β1, which have been implicated in curbing trophoblast migration and proliferation [2,14,19]. This elevated PLAC8 expression could potentially function as an interplay molecule between signaling pathways, forming a molecular bridge connecting placental hypoxia to downstream mediators associated with preeclampsia [25,42]. This suggests that O_2_ concentration and TGF-β1 synergistically regulate PLAC8 expression, potentially linking placental hypoxia to downstream mediators of preeclampsia. Other several factors linked to placentation defects have been studied, encompassing chronic inflammation, oxidative stress, and altered levels of angiogenic factors, such as vascular endothelial growth factor (VEGF) and placental growth factor (PGF), in addition to elevated levels of anti-angiogenic factors [2,39,43,44]. Our results significantly underscore that *PLAC8* overexpression was associated with a decline in the expression of VEGF and PGF (as depicted in Figure 5). This interaction could facilitate communication with angiogenic-promoting factors, which are responsible for orchestrating placental neovascularization. The mechanisms underpinning these defects remain intricate and incompletely understood. However, various signaling pathways appear to be involved. One potential avenue is the Notch signaling pathway, which plays a role in governing cell cycling, differentiation, and invasion. It is postulated that PLAC8 might interact with the Notch pathway to modulate the expression of angiogenic factors, thereby influencing placental vascularization [45]. Another plausible pathway is the epithelial-to-mesenchymal transition (EMT), a cellular process wherein epithelial cells transform into more motile and invasive mesenchymal cells. Remarkably, PLAC8 has been demonstrated to hinder EMT, potentially acting as a safeguard against the emergence of placental defects [46]. These findings underscore the intricate web of interactions through which PLAC8 might exert its influence on cellular processes central to placental development. Although our study did not explore these pathways experimentally, the phenotypic changes observed are consistent with their potential involvement. Further mechanistic studies are warranted to delineate how PLAC8 may interact with Notch signaling and EMT processes to regulate trophoblast differentiation and placental function. In conclusion, this study provides strong evidence that PLAC8 plays a crucial role in trophoblast function and placental development. Specifically, *PLAC8* overexpression in trophoblast cells enhances invasiveness but disrupts tubular structure formation, mirroring its role in endothelial-like trophoblast differentiation, essential for proper placentation. This suggests potential implications of *PLAC8* overexpression in placental disorders, like preeclampsia, and other trophoblastic diseases, such as hyperinvasive placentas acretas, incretas, and percretas. Additionally, PLAC8’s interaction with genes linked to vasculogenesis, such as *TGF-β1, VEGFA*, and *PGF*, indicates an intricate molecular pathway that warrants further investigation. Understanding this interplay is vital for elucidating the mechanisms of placental vascularization and its impact on pregnancy outcomes.

Some limitations of this study deserve to be mentioned. This study utilized a single first-trimester human cell line (Swan-71), which may not fully capture the variability in cellular responses. The findings are based on in vitro experiments, and their applicability to in vivo conditions remains uncertain. Furthermore, the study focused solely on the effects of *PLAC8* knockdown and overexpression in trophoblast cells, which may differ in other cell types, such as endothelial cells. The long-term effects of PLAC8 overexpression were not explored, highlighting the need for further research to understand its dynamic impact over time.

## 4. Materials and Methods

### 4.1. Reagents and Plasmid Constructs

Plasmid constructs, namely pRP[Exp]-Bsd-CMV>hPLAC8 (ID: VB200131-1104sgx) and pRP[shRNA]-Bsd-U6>hPLAC8[shRNA#1] (ID:VB200131-1108stk), of the PLAC8 gene were constructed and packaged by VectorBuilder, Inc. (VectorBuilder, San Mateo, CA, USA).

### 4.2. Cell Culture

The human first-trimester trophoblast cell lines Swan-71 (ATCC, Gaithersburg, Maryland, USA) were cultured in RPMI-1640 medium (Gibco Life Technologies, Grand Island, NY, USA) supplemented with 10% fetal bovine serum (FBS) (Gibco Life Technologies, NY, USA), 100 mg/mL streptomycin (Corning Life Science, Tewksbury, MA, USA), and 100 Units/mL penicillin (Corning Life Science, MA, USA) and maintained in an atmosphere containing 5% CO_2_ at 37 °C.

### 4.3. Cell Transfection

Swan-71 cells were transiently transfected with the PLAC8 overexpression plasmid construct (pRP[Exp]-Bsd-CMV>hPLAC8); meanwhile, PLAC8 silencing was achieved by transfection with the short hairpin plasmid construct (pRP[shRNA]-Bsd-U6>hPLAC8[shRNA#1]). Transfection was carried out with Lipofectamine 3000 (Invitrogen, Waltham, CA, USA) following the manufacturer’s instructions. The transfection of Swan-71 cells was shown to overexpress and knockdown the PLAC8 mRNA through the evaluation of the relative protein abundancy.

### 4.4. Cell Viability

Swan-71 cell viability was assessed using the MTT (3-(4,5-dimethylthiazol-2-yl)-2,5-diphenyltetrazolium bromide) method. Seventy-two hours after transfection, cells were harvested with 0.25% trypsin, and 10,000 cells/well were seeded in 96-well microplates. Monolayers were incubated with 100 µL MTT (0.5 mg/mL) for 4 h (37 °C, 5% CO_2_). After incubation, the formazan salts were dissolved with dimethyl sulfoxide for 15 min. Absorbance at 575 nm was measured using an Infinite M200 Pro microplate reader (Bio-Tec, Forest Lake, MN, USA).

### 4.5. Invasion Assay

For the invasion assay, the Swan-71 cells were resuspended in serum-free culture medium RPMI-1640 (106 cells/mL) and seeded (200 µL) in transwells containing polycarbonate filters (8 µm pore) (EMD, Gibbstown, NJ, USA). The transwells were pre-coated with BD-Matrigel (3 mg/mL) (BD Biosciences, Franklin Lakes, NJ, USA) and incubated (37 °C, 5% CO_2_, 1 h) to allow polymerization. RPMI-1640 medium (500 µL) supplemented with 10% FBS was added to the lower chamber of the transwells and incubated for 24 h (37 °C, 5% CO_2_). At the end of the invasion assay, cells attached to the transwells were washed (2×) with PBS 1%, fixed in 4% paraformaldehyde (Sigma-Aldrich, Billerica, MA, USA), and stained (20 min, room temperature) with 0.2% crystal violet (Sigma-Aldrich, MA, USA). Transwells were washed with PBS to remove the excess of crystal violet stain. Non-invading cells on the upper part of the inserts were gently removed with a cotton swab embedded in PBS. Cells that migrated to the underside of the inserts were photographed under a phase-contrast inverted light microscope (Olympus CKX53; Tokyo, Japan) equipped with a digital microscope camera (Moticam 3 (Motic, Kowloon Bay, Hong Kong)). Cells were then lysed for 10 min in 300 µL lysis buffer (100 mmol/L NaCl, SDS 1%, 50 mmol/L Tris/HCl, pH 7.4) at room temperature. Lysed cells were analyzed using an Infinite M200 Pro microplate reader (Tecan, Austria, GmbH, Grödig, Austria) at an absorbance of 595 nm.

### 4.6. Differentiation Assay

The endothelial-like differentiation of the Swan-71 cells was achieved as follows. First, 770,000 cells per well were seeded into 6-well plates pre-coated with 550 μL of growth factor-reduced Matrigel (BD Biosciences, Franklin Lakes, NJ, USA), or uncoated plastic wells (for the control), with 2 mL serum-reduced media (SRM, RPMI-1640 supplemented with 5% FBS, 100 mg/mL streptomycin, and 100 units/mL penicillin) and incubated in 20% O_2_ and 5% CO_2_ for 24 h, as per the manufacturer’s instructions. Cells were photographed using an Olympus IX71 inverted microscope (Olympus Corporation, Tokyo, Japan) with a Moticam 3.

### 4.7. Effect of Oxygen and TGFb on Trophoblast Invasion

Swan-71 cells were maintained for 24 h at 37 °C in RPMI 1640 medium supplemented with 10% FBS under an atmosphere of 5% CO_2_-balanced N2 and either 20%, 8%, or 1% O_2_ in an automated hypoxia chamber. The media were changed for RPMI 1640 without FSB for 6 h and supplemented with Ang II 100 nM and 2500 nM (Sigma-Aldrich, St. Louis, MA, USA) and TGFb 1 nM and 10 nM (ABCAM) and then cultured 24 h in the same O_2_ conditions. RNA and protein extractions were performed for each sample.

### 4.8. Protein Extraction and Western Blotting

Swan-71 cell protein extracts were obtained after washing the cells (×2) with ice-cold PBS (4 °C); cells were harvested in 300 µL of lysis buffer (100 mmol/L NaCl, 0.5% Triton X-100, 1% SDS, 50 mmol/L Tris/HCl, pH 7.4), containing a mixture of protease inhibitors (0.3 µmol/L aprotinin, 1 mmol/L ethylenediaminetetraacetic acid (EDTA), 1 µmol/L leupeptin, and 1 mmol/L phenylmethylsulfonyl fluoride (PMSF) (Thermo-Fisher Scientific, Waltham, MA, USA)). Extracts were sonicated for 20 s in a handheld tissue homogenizer at 4 °C, and then stored at −20 °C. Aliquots of each sample were used to determine the concentration of total protein by the bicinchoninic acid with a protein assay kit (micro BCA) (Thermo-Fisher Scientific, Waltham, MA, USA) according to the manufacturer’s instructions. Total protein extracts were adjusted to 30 µg and separated by polyacrylamide gel (15%) electrophoresis (SDS-PAGE) and then transferred to P polyvinylidene difluoride membranes (BioRad, Hercules, CA, USA). The membranes were incubated for 1 h with Tris buffer saline–Tween 20 (TBS-T) containing 5% non-fat dry milk. Proteins were probed with primary polyclonal rabbit anti-PLAC8 (1:500 dilution, 18 h, 4 °C) (ABCAM ab122652), while monoclonal rabbit anti-ß-actin (1:5000 dilution, 18 h, 4 °C) (Sigma-Aldrich) and monoclonal rabbit anti-GAPDH (1:5000 dilution, 18 h, 4 °C) (Sigma-Aldrich) in TBS-T 5% non-fat dry milk were used as loading controls. The membranes were rinsed in TBS-T 5% non-fat dry milk and further incubated (1 h) in a solution containing secondary horseradish peroxidase-conjugated goat anti-rabbit antibodies (Thermo Scientific, CA, USA). Proteins were detected by enhanced chemiluminescence using an ImageQuant LAS 500 chemiluminescence CCD camera (CYTIVA, New Haven, CT, USA) and then quantified by densitometry.

### 4.9. RNA Extractions and Gene Expression RT-PCR

Total RNA from differentiated trophoblast cells was extracted essentially as previously described [47,48]. Briefly, tube-like structures were recovered from Matrigel using EDTA extraction in cold PBS, followed by multiple washing steps with ice-cold PBS and centrifugation. The final cell pellet was resuspended in the lysis buffer provided in the E.Z.N.A. RNA I Kit (Omega Bio-tek, Norcross, GA, USA), and total RNA was subsequently extracted according to the manufacturer’s instructions. One microgram of each total RNA preparation was then subjected to reverse transcription using SuperScript™ II Reverse Transcriptase (Invitrogen, Carlsbad, CA, USA). This extraction and conversion procedure was applied to Swan-71 cells that had undergone genetic modifications, either for PLAC8 overexpression or knockdown. Subsequently, the extracted RNA samples underwent analysis through real-time PCR methodology. Real-time RT-PCR was executed within a total volume of 20 μL, utilizing the Takyon™ Rox SYBR^®^ QPCR master mix (Eurogenet, Seraing Belgium). Relative expression levels were calculated using the—ΔΔCt method, normalized to those of the housekeeping gene, glyceraldehyde-3-phosphate dehydrogenase (GAPDH). The oligonucleotides used were synthesized by ITD (Integrated DNA Technologies, Coralville, IA, USA). The focus of the gene expression analysis was specifically aimed at genes associated with the promotion of vasculogenesis, including well-established factors, such as VEGF and PGF. Additionally, the analysis encompassed the evaluation of genes acting as inhibitors of angiogenesis, with particular emphasis on ANGPT2 (outlined conditions and oligonucleotides are in Table 1).

### 4.10. Statistical Analysis

Data are presented as mean ± standard deviation (SD), represented as 3 replicates per experimental point. The normality of the data was determined with Kolmogorov–Smirnov’s test. Comparisons between two groups were performed by Student’s T test and the Mann–Whitney U test for non-parametric data. The differences between more than two groups were determined by Friedman’s test for non-parametric data. If Friedman’s test demonstrated a significant interaction between variables, post hoc analyses were performed by the Dunns’s correction test. The statistical software GraphPad Prism 7.0c (Boston, MA, USA) was used for data analysis.

## Figures and Tables

**Table 1:** Oligonucleotide sequences for gene expression.

Gene	Exon	Oligonucleotides	Sequence	Tm	Cycles
*PLAC8*	3–4	PLAC8-F PLAC8-R	5′-aactccaactggcagacagg-3′ 5′-gccatatcgggtcctgtaga-3′	60 °C	40
*GAPDH*	1	GAPDH-F GAPDH-R	5′-aggtcggtgtgaacggatttg-3′ 5′-tgtagaccatgtagttgaggtca-3′	60 °C	40
*ANGPT2*	7–8	ANGPT2-F ANGPT2-R	5′-ccccactgttgctaaagaaga-3′ 5′-catcctcacgtcgctgaata-3′	60 °C	40
*PGF*	3–4	PGF-F PGF-R	5′-gtggacgtcgtgtccgagta-3′ 5′-aacgtgctgagagaacgtca-3′	60 °C	40
*VEGF*	2–3	VEGF-F VEGF-R	5′-aaggaggagggcagaatcat-3′ 5′-aagatgtccaccagggtctc-3′	60 °C	40

PLAC8: placenta-specific 8, GAPDH: glyceraldehyde-3-phosphate dehydrogenase, ANGPT2: angiopoietin-2, PGF: placental growth factor, VEGF: vascular endothelial growth factor.

## Data Availability

No new data were created or analyzed in this study.

## Abbreviations

The following abbreviations are used in this manuscript:

PLAC8	Placenta-Specific 8
GAPDH	Glyceraldehyde-3-phosphate dehydrogenase
ANGPT2	Angiopoietin-2
PGF	Placental growth factor
VEGF	Vascular endothelial growth factor
